# Bifunctional 4,5-Diiodoimidazole Interfacial Engineering Enables Simultaneous Defect Passivation and Crystallization Control for High-Efficiency Inverted Perovskite Solar Cells

**DOI:** 10.3390/nano15100766

**Published:** 2025-05-20

**Authors:** Huaxi Gao, Yu Zhang, Ihtesham Ghani, Min Xin, Danish Khan, Junyu Wang, Di Lu, Tao Cao, Wei Chen, Xin Yang, Zeguo Tang

**Affiliations:** 1 School of Energy and Environmental Sciences, Yunnan Normal University, Juxian Road 768, Kunming 650500, China; 2223160030@ynnu.edu.cn (H.G.); 2223160035@ynnu.edu.cn (M.X.); 2 The College of New Materials and New Energies, Shenzhen Technology University, Lantian Road 3002, Shenzhen 518118, China; zhangyu1@sztu.edu.cn (Y.Z.); khandanish@sztu.edu.cn (D.K.); 3 The College of Materials Science and Engineering, Beijing University of Technology, 100 Pingleyuan, Beijing 100124, China; 4 College of Engineering Physics, Shenzhen Technology University, Shenzhen 518118, China; caotao@stu.hubu.edu.cn (T.C.); chenwei@sztu.edu.cn (W.C.)

**Keywords:** perovskite solar cell, imidazole, buried interface, high efficiency

## Abstract

Despite the rapid efficiency advancement of perovskite solar cells (PSCs), non-radiative recombination at the buried interface between self-assembled monolayers (SAMs) and perovskite remains a critical bottleneck, primarily due to interfacial defects and energy level mismatch. In this study, we demonstrate a bifunctional interlayer engineering strategy by introducing 4,5-diiodoimidazole (4,5-Di-I) at the Me-4PACz/perovskite interface. This approach uniquely addresses two fundamental limitations of SAM-based interfaces: the insufficient defect passivation capability of conventional Me-4PACz due to steric hindrance effects and the poor perovskite wettability on hydrophobic SAM surfaces that exacerbates interfacial voids. The imidazole derivatives not only form strong Pb–N coordination bonds with undercoordinated Pb^2+^ but also modulate the surface energy of Me-4PACz, enabling the growth of pinhole-free perovskite films with preferential crystal orientation. The champion device with 4,5-Di-I modification achieves a power conversion efficiency (PCE) of 24.10%, with a V_OC_ enhancement from 1.12 V to 1.14 V, while maintaining 91% of initial PCE after 1300 h in N₂ atmosphere (25 °C), demonstrating superior stability under ISOS-L-2 protocols. This work establishes a universal strategy for interfacial multifunctionality design, proving that simultaneous defect suppression and crystallization control can break the long-standing trade-off between efficiency and stability in solution-processed photovoltaics.

## 1. Introduction

According to the IEA 2025 Energy Outlook Report, global energy demand is now projected to surge by 52% by 2050, requiring photovoltaic installations to scale exponentially from 1.5 TW in 2023 to 85 TW by mid-century for net-zero compliance [1]. Perovskite solar cells (PSCs) are positioned as a cornerstone of this transition, offering a disruptive thin-film technology capable of achieving over 30% efficiency in industrial tandem configurations with potential <$0.30/W module costs—a critical threshold for terawatt-scale deployment [2]. Recent advances in blade-coated monolithic perovskite/silicon tandem solar cells have achieved a certified power conversion efficiency of 31.2% through interfacial 2D/3D heterojunction engineering [3]. Single-junction PSCs have achieved a certified quasi-steady-state PCE of 26.8% [4], rivaling monocrystalline silicon cells. This rapid progress stems from perovskites’ unique optoelectronic properties: tunable bandgap (1.2–2.3 eV via halide alloying) [5], long carrier diffusion length [6], high optical absorption coefficient [7], small exciton binding energy [8], high carrier mobility [9], relatively balanced electron and hole transfer [10], and solution processability at <150 °C [11].

Despite these advances, PSCs still fall short of IEC 61215 standards requiring >1000 h T80 under 85 °C/85% RH damp heat testing (IEC 61215:2023 Clause 10.21) [12]. The efficiency–stability trade-off predominantly originates from interfacial ion migration (activation energy ≈ 0.5 eV) [13] and defect-assisted degradation at buried interfaces [14]. Buried interfaces between charge transport layers and perovskites exhibit defect densities up to 10^1^⁶ cm^−3^, accounting for >60% of non-radiative recombination losses [15,16]. Self-assembled monolayers (SAMs) like [4-(3,6-Dimethyl-9H-carbazol-9-yl) butyl] phosphonic acid (Me-4PACz) have revolutionized hole transport in inverted PSCs by enabling sub-1 nm thick charge extraction layers with tunable energy levels (−5.34 eV HOMO) and low-temperature processability (<100 °C) [17]. However, their inherent hydrophobicity (water contact angle = 92° ± 3°) induces perovskite precursor dewetting, creating 10–50 nm voids. Recent studies show that SAM micelle formation during spin-coating generates nanoscale pinholes that accelerate Ag electrode corrosion via iodide ion migration [18]. Current passivation strategies (e.g., alkylammonium salts) fail to simultaneously address wettability and defect passivation due to steric hindrance effects [19,20].

Herein, we design a bifunctional 4,5-diiodoimidazole (4,5-Di-I) interlayer that covalently anchors to Me-4PACz via I···H-C interactions, reducing water contact angle to 36° ± 2°, while passivating undercoordinated Pb^2+^ via N→Pb coordination. This dual-action mechanism suppresses interfacial recombination (τₐᵥₑ from 978 ns to 1456 ns) and enables [100]-oriented perovskite growth with smaller porosity, achieving 24.10% PCE with T80 > 1300 h under ISOS-L-2 protocols (N₂ atmosphere, 65 °C, 1-sun equivalent LED illumination).

## 2. Experimental Section

### 2.1. Materials

4,5-diiodoimidazole were purchased from Shanghaiyuanye Bio-Technology Co., Ltd. (Shanghai, China) phenethylammonium chloride (PEACl, 99.5%), (4-(3,6-dimethyl-9H-carbazole-9-yl) butyl) phosphonic acid (Me-4pacz, 99.5%), formamidinium iodide (FAI, 99.9%), methylammonium iodide (MAI, 99.5%), methylammonium Chloride (MACl, 99.9%), cesium iodide (CsI, 99.9%), lead iodide (PbI_2_, 99.999%), Fullerene (C60, 99.9%), and 2,9-dimethyl-4,7-diphenyl-1,10-phenanthroline (BCP, 98%) were bought from Xi’an Polymer Light Technology (Xi’an, China). NiO_x_ nanoparticles were procured from Advanced Election Technology (Yingkou, China). Chlorobenzene (CB, anhydrous,99.8%), N,N-dimethylformamide (DMF, anhydrous, 99.8%), isopropyl alcohol (IPA), and dimethyl sulfoxide (DMSO, anhydrous, ≥99.9%), were obtained from Sigma-Aldrich (St. Louis, MO, USA).

### 2.2. Device Fabrication

FTO glass was sequentially cleaned using an alkaline glass cleaner, deionized water, isopropanol (IPA), and ethanol under ultrasonication for 20 min each, followed by drying with a nitrogen gun and UV-ozone treatment for 15 min each. NiO_x_ dispersion (10 mg/mL in deionized water) was sonicated for 10 min, and 80 μL of the dispersion was spin-coated onto the FTO substrate at 2000 rpm for 30 s, followed by annealing at 150 °C for 30 min in ambient air. The substrate was then immediately transferred into a nitrogen-filled glovebox, where 100 μL of a Me-4PACz solution (0.5 mg/mL in ethanol) was spin-coated at 4000 rpm for 30 s and annealed at 100 °C for 10 min. Subsequently, 80 μL of a 4,5-diiodoimidazole solution (0.5 mg/mL) was spin-coated at 4000 rpm for 30 s and annealed at 70 °C for 5 min.

To prepare a 1.5 M Cs_0.05_FA_0.85_MA_0.1_PbI_3_ perovskite precursor solution, CsI (19.5 mg), FAI (219.3 mg), MAI (23.8 mg), PbI_2_ (760.7 mg), and MACl (12.66 mg) were dissolved in 1 mL of a mixed solvent of DMF and DMSO (volume ratio 4:1). The solution was stirred at 60 °C for at least 3 h.

The perovskite solution was spin-coated at 1000 rpm for 10 s and then at 4000 rpm for 40 s, with 160 μL of CB antisolvent dripped 5 s before the end of the spin-coating process. The film was annealed at 100 °C for 30 min in the nitrogen-filled glovebox. A PEACl solution in IPA was then spin-coated at 4000 rpm for 30 s. Finally, 30 nm of C60 and 6 nm of BCP were thermally evaporated under a vacuum of 1 × 10^−5^ Pa, followed by the deposition of 100 nm of Ag at 1 × 10^−4^ Pa.

## 3. Results and Discussion

**Figure 1:**
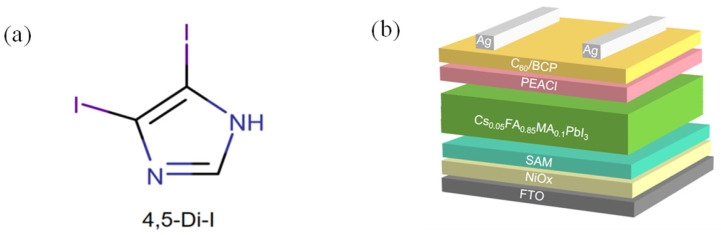
(**a**) Chemical structure of 4,5-Di-I; (**b**) structure of PSC device.

To construct the interfacial layer, 4,5-diiodoimidazole [chemical structure shown in Figure 1a was dissolved in DMF and spin-coated onto the NiO_x_/Me-4PACz hole transport layer (HTL). The resulting device architecture is illustrated in Figure 1b.

**Figure 2:**
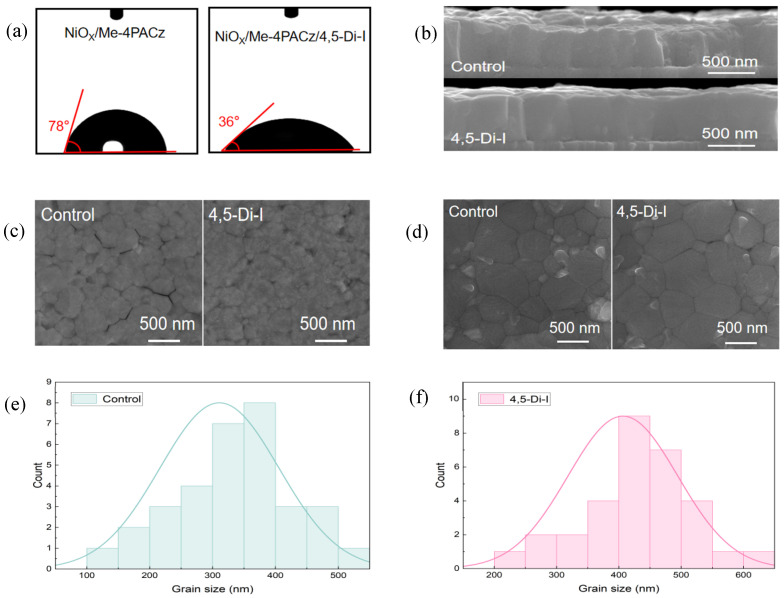
(**a**) Water contact angle of different HSLs. (**b**) Cross-section SEM images of perovskite films on different SAMs. (**c**) SEM images of different SAM treated perovskite buried interface. (**d**) SEM image of perovskite surface. Grain size distribution of (**e**) control and (**f**) 4,5-Di-I, which are based on the Gaussian distribution.

Water-contact-angle measurements are shown in Figure 2a; they reveal that the imidazole modification significantly enhances the wettability of the SAM layer, reducing the contact angle from 78° (pristine Me−4PACz) to 36°. This improved wettability enables complete and uniform coverage of the perovskite precursor solution on the SAM layer, creating an ideal substrate for the growth of high-quality perovskite films with minimized interfacial voids. As shown in Figure 2b, the 4,5-Di-I-modified perovskite film exhibits superior structural integrity compared to the control. Specifically, the modified film shows a more uniform thickness with consistent height across the layer, which contributes to the formation of a smoother and flatter perovskite surface. In contrast, the control sample displays a non-uniform thickness, leading to surface roughness and local height variations. These irregularities may negatively affect light absorption and carrier transport and hinder the subsequent deposition of the electron transport layer. In cases where surface protrusions are too pronounced, the electron transport layer may fail to achieve complete coverage, resulting in severe leakage paths. Additionally, the grains in the 4,5-Di-I-modified film are more vertically aligned and exhibit fewer misorientations. Such vertical grain alignment reduces the number of grain boundaries along the carrier transport direction, thereby minimizing recombination centers. It also promotes more uniform energy band alignment across the film, improving interfacial charge extraction and contributing to enhanced open-circuit voltage. Furthermore, the lateral grain size in the modified film is larger and more uniform, which further reduces grain boundary density and facilitates efficient charge transport toward the electrodes, leading to enhanced carrier mobility.

To further investigate the buried interface morphology, a peeling technique was employed. As shown in Figure 2c, the buried interface of the control sample reveals numerous gaps between grains, likely due to poor wetting. These nanoscale voids act as recombination sites and hinder efficient hole extraction. In contrast, the 4,5-Di-I-modified film exhibits a much more compact interfacial structure with tightly connected grains, resulting in a denser film that favors charge extraction. Top-surface SEM analysis (Figure 2d) confirms that imidazole treatment enhances grain coalescence, increasing average grain size from 317 ± 20 nm (control) to 406 ± 20 nm, while reducing grain boundary density by 28%, Figure 2e,f. These microstructural improvements collectively minimize carrier trapping at interfacial and grain boundary defects, directly correlating with enhanced device performance.

**Figure 3:**
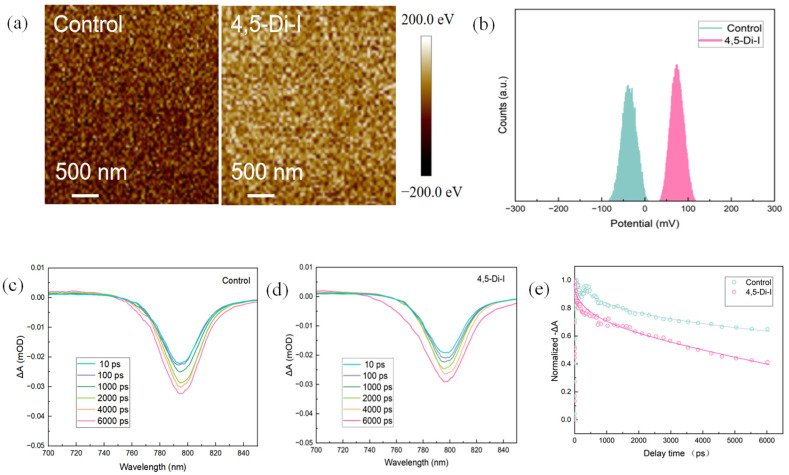
(**a**) KPFM images of control and 4,5-Di-I. (**b**) Potential of control and 4,5-Di-I. The delay time-dependent TA spectra of (**c**) control and (**d**) 4,5-Di-I. (**e**) corresponding normalized decay kinet.

Kelvin probe force microscopy (KPFM) reveals that 4,5-diiodoimidazole modification significantly modulates the surface potential landscape. Figure 3a shows a 3 × 3 μm^2^ potential map where the modified film exhibits a 32% higher average surface potential (ΔΨ = +0.13 eV) than the control, indicating enhanced p-type character for improved hole extraction. The potential distribution histogram (Figure 3b) demonstrates reduced potential fluctuation (standard deviation: 8 mV vs. 18 mV in control), confirming superior interfacial homogeneity.

Femtosecond transient absorption spectroscopy (fs-TAS) was employed to study the ultrafast carrier dynamics in both 4,5-diiodoimidazole (4,5-Di-I)-modified and pristine perovskite films (Figure 3c–e). The 4,5-Di-I-modified perovskite exhibited faster ground-state bleach (GSB) decay dynamics. Following the approach reported by Serpetzoglou et al., we fitted the transient GSB decay using a tri-exponential model based on Equation (1) [21]:

(1) $$y=y_{0}+\omega_{1}\exp\left(-\frac{x}{\tau_{1}}\right)+\omega_{2}\exp\left(-\frac{x}{\tau_{2}}\right)+\omega_{3}\exp\left(-\frac{x}{\tau_{3}}\right)$$

*τ_1_* corresponds to the carrier trapping time at grain boundaries and the HTL interface, *τ_2_* denotes the hole transfer time from the perovskite to the HTL, *τ_3_* represents the free carrier recombination lifetime.

As shown in Table 1, the 4,5-Di-I-modified film exhibited shorter *τ_1_* and *τ_2_* values (*τ_1_* = 3.3 ps vs. 5.1 ps in the control; *τ_2_* = 382 ps vs. 456 ps in the control), indicating faster trap filling and more efficient free carrier injection, which result in enhanced electrical performance of the perovskite layer. These results suggest more effective charge transfer from the perovskite to the transport layer. The suppressed hole accumulation is consistent with the observed improvement in V_OC_ (from 1.12 V to 1.14 V) and fill factor (from 81.4% to 84.5%).

In addition to exponential fitting, we analyzed recombination dynamics using a polynomial rate equation based on Equation (2) [22]:

(2) $$dn(t)dt=-k_{3}n^{3}-k_{2}n^{2}-kn$$

*k_1_* represents trap-assisted recombination, *k_2_* is the bimolecular recombination rate, and *k_3_* corresponds to Auger (three-body) recombination.

Our analysis focused on *k_2_*, which characterizes free carrier recombination and is a critical parameter for solar cell performance. As shown in Table 2, the 4,5-Di-I-modified film exhibited a higher *k_2_* value (3.9 × 10^−10^ cm^3^·s^−1^) compared to the control (2.6 × 10^−10^ cm^3^·s^−1^), indicating a longer carrier diffusion length and improved perovskite film quality in the 4,5-Di-I-modified device.

Table 1 summarizes the exponential fitting results for the two device architectures.

**Table 1:** Exponential fitting results of the fs-TAS.

	Temperature (K)	λ_max_ (nm)	τ_1_ ± 2 (ps)	τ_2_ ± 3 (ps)	τ_3_ ± 8 (ps)
Control	300	780	5.1	456	1018
4,5-Di-I	300	780	3.1	382	1022

Table 2 presents the corresponding recombination coefficients extracted from polynomial fitting.

**Table 2:** Polynomial fitting results of the recombination curves.

	λ_max_ (nm)	K_3_ (cm^6^s^−1^) ± 0.4	K_2_ (cm^3^s^−1^) ± 0.2	K_1_ (μs^−1^) ± 0.1
Control	780	8.3 × 10^−15^	2.6 × 10^−10^	4.5 × 10^−5^
4,5-Di-I	780	7.7 × 10^−15^	3.9 × 10^−10^	2.9 × 10^−5^

**Figure 4:**
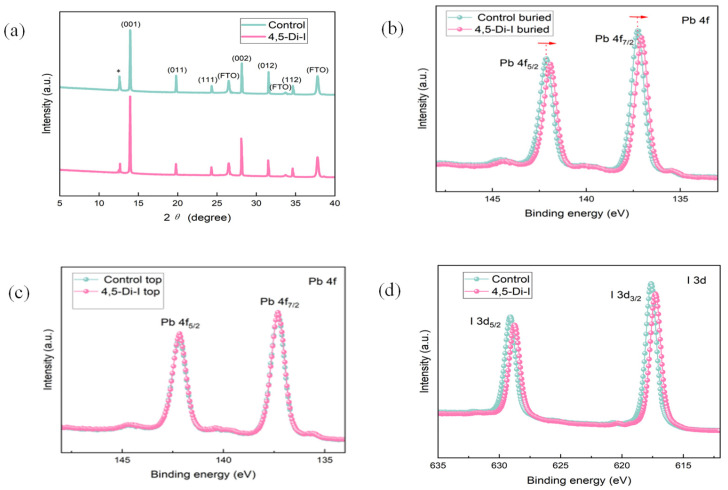
(**a**) The XRD patterns of perovskites (* indicates PbI_2_). (**b**) XPS spectra of Pb 4f at the buried interface of control and 4,5-Di-I perovskite films (the direction of the arrows represents the shift direction of the XPS spectra). (**c**) XPS spectra of Pb 4f at the top interface of control and 4,5-Di-I perovskite films. (**d**) XPS spectra I 3d of control and 4,5-Di-I perovskite films.

X-ray diffraction (XRD) analysis (Figure 4a) confirms that the imidazole-modified perovskite maintains phase purity while exhibiting stronger (001)-oriented crystallinity (FWHM: 0.18° vs. 0.24° in control), consistent with the observed grain size enlargement in SEM. X-ray photoelectron spectroscopy (XPS) at the buried interface reveals a 0.3 eV downward shift in Pb 4f binding energy (Figure 4b), directly evidencing N→Pb^2+^ coordination that passivates undercoordinated lead defects. No such shift occurs at the top interface (Figure 4c), confirming the localized nature of imidazole’s interfacial modification.

This coordination increases electron density around Pb nuclei through lone-pair electron donation from imidazole’s nitrogen atoms to Pb^2+^’s empty 6p orbitals, thereby reducing Pb-related defect states. Additionally, the characteristic peak of I 3d shifts toward lower binding energy, indicating a reduction in I vacancies (Figure 4d). The suppressed defect formation directly correlates with enhanced V_OC_ and reduced non-radiative recombination.

**Figure 5:**
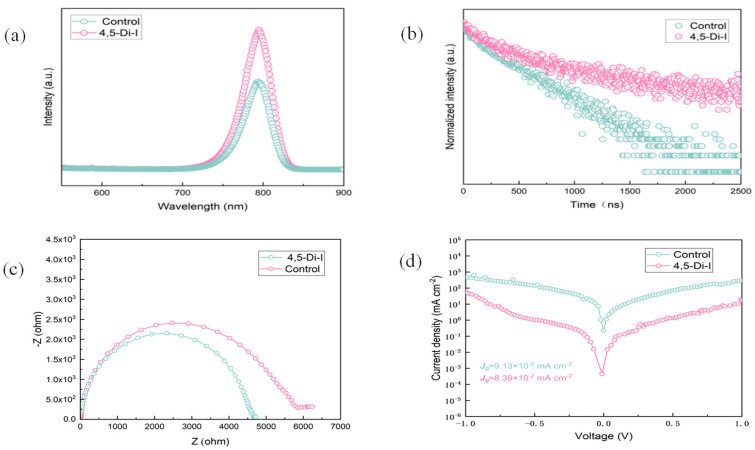
(**a**) PL spectra of perovskite. (**b**) TRPL spectra of perovskite. (**c**) Nyquist plots. (**d**) Dark J–V curves.

Photoluminescence (PL) spectroscopy reveals a 1.7-fold enhancement in emission intensity for imidazole-modified films (Figure 5a), directly indicating reduced trap-state density and suppressed non-radiative recombination. Time-resolved PL (TRPL) measurements (Figure 5b) quantify this improvement, showing a prolonged average carrier lifetime (τₐᵥₑ = 1456 ns vs. 978 ns for control), which we attribute to effective passivation of undercoordinated Pb^2+^ and halide vacancies via N→Pb coordination—consistent with the XPS-observed 0.3 eV Pb 4f binding energy shift. Nyquist plots of electrochemical impedance (Figure 5c) further corroborate these findings: the imidazole-modified device exhibits increased recombination resistance, confirming fewer interfacial defect states. These results collectively demonstrate that 4,5-diiodoimidazole minimizes carrier trapping, while enhancing charge transport efficiency.

Electrochemical impedance spectroscopy (EIS) measurements were conducted under dark conditions with an applied bias of 1.0 V. The impedance data were fitted using an equivalent circuit model consisting of a series resistance (Rs), a recombination resistance (Rrec), and a capacitor (C), assuming an ideal interfacial capacitance. The overall impedance behavior was simulated using the following expression:

(3) $$Z(\omega )=R_{S}+\frac{1}{\frac{1}{R_{\mathrm{rec}}}+j\omega C}$$

In Equation (3), Rs is the series resistance (Ω), Rrec is the recombination resistance (Ω), j is the imaginary unit, ω is the angular frequency (rad·s^−1^), and C is the capacitance (F).

EIS reveals a 21.6% increase in recombination resistance for the imidazole-modified device (Rrec = 5846 Ω vs. 4783 Ω for control; Figure 5c and Table 3), directly evidencing reduced interfacial defect density through N→Pb^2+^ coordination. This suppression of non-radiative recombination correlates with the observed V_OC_ enhancement from 1.12 V to 1.14 V. Dark current measurements (Figure 5d) further confirm improved interfacial integrity: the modified devices exhibit 63% lower leakage current at −0.5 V bias, indicating suppressed shunt paths and enhanced charge selectivity [23]. These complementary AC/DC analyses conclusively link the imidazole-induced interface optimization to simultaneous improvements in V_OC_, FF, and PCE.

**Figure 6:**
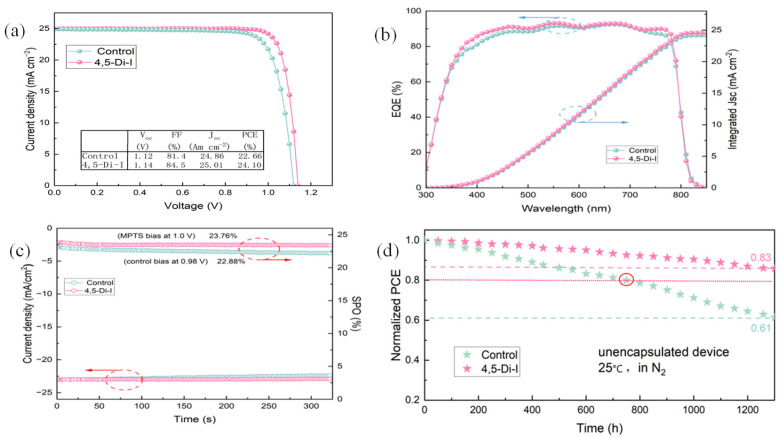
(**a**) J−V curve of the best-performing control and 4,5-Di-I-treated device. (**b**) EQE and integrated JSC. (**c**) The SPO of the control and 4,5-Di-I. (**d**) Normalized PCE of unencapsulated devices in N_2_ atmosphere.

As shown in Figure 6, the 4,5-diiodoimidazole-modified device achieves an improved PCE of 24.10% (V_OC_ = 1.14 V, J_SC_ = 25.01 mA/cm^2^, FF = 84.5%), representing a 1.44% absolute efficiency gain over the control (22.66%, V_OC_ = 1.12 V, J_SC_ = 24.86 mA/cm^2^, FF = 81.4%). J-V curves in Figure 6a and EQE spectra in Figure 6b confirm superior performance, with the modified device showing a 1.7% higher integrated J_SC_ (24.57 vs. 24.15 mA/cm^2^). Maximum power point tracking (MPPT) under continuous 1-sun illumination [Figure 6c] demonstrates exceptional operational stability: the modified device retains 98.6% of its initial PCE (23.76% stabilized) after 325 s, outperforming the control device (22.88% stabilized). Long-term aging tests in N₂ (25 °C, ISOS-L-1 protocols) reveal a T80 of 1300 h for the modified device (83% PCE retention) versus T80 = 740 h for the control [61% PCE retention; Figure 6d]. These results conclusively demonstrate that 4,5-diiodoimidazole modification achieves simultaneous efficiency and stability enhancements through interfacial defect suppression and carrier dynamics optimization.

## 4. Conclusions

By synergistically addressing perovskite wettability and interfacial defects, this work demonstrates that 4,5-diiodoimidazole (4,5-Di-I) modification significantly enhances the performance of Me-4PACz-based inverted perovskite solar cells. The iodine-rich derivative reduces the water contact angle of Me-4PACz from 78° to 36°, enabling uniform perovskite nucleation and eliminating interfacial voids, while its N→Pb^2+^ coordination (0.3 eV XPS shift) passivates defects, suppressing non-radiative recombination (TRPL: 1456 ns vs. 978 ns). This dual-action strategy yields a drastically improved PCE of 24.10% (V_OC_ = 1.14 V, FF = 84.5%) with T80 > 1300 h in N₂, outperforming control devices in both efficiency (1.44% absolute gain) and stability (T80 = 740 h). These advances establish a universal framework for SAM-based interface engineering, where molecular design simultaneously optimizes crystallization dynamics and defect thermodynamics. Future efforts will target iodine substitution effects and eco-friendly lead-leakage mitigation to accelerate industrial adoption.

## Figures and Tables

**Table 3:** Fitted values of EIS measurement from the equivalent circuit components.

	Rs (Ω) ± SD	Rrec (Ω) ± SD	C (nF) ± SD
Control	29.3 ± 0.3	4783 ± 60	34.2 ± 0.6
4,5-Di-I	31.5 ± 0.2	5846 ±45	29.8 ± 0.6

## Data Availability

The original contributions presented in this study are included in the article. Further inquiries can be directed to the corresponding author.
